# Visceral Adiposity Index: An Indicator of Adipose Tissue Dysfunction

**DOI:** 10.1155/2014/730827

**Published:** 2014-04-14

**Authors:** Marco Calogero Amato, Carla Giordano

**Affiliations:** Section of Cardio-Respiratory and Endocrine-Metabolic Diseases, Biomedical Department of Internal and Specialist Medicine (DIBIMIS), University of Palermo, Piazza delle Cliniche 2, 90127 Palermo, Italy

## Abstract

The Visceral Adiposity Index (VAI) has recently proven to be an indicator of adipose distribution and function that indirectly expresses cardiometabolic risk. In addition, VAI has been proposed as a useful tool for early detection of a condition of cardiometabolic risk before it develops into an overt metabolic syndrome. The application of the VAI in particular populations of patients (women with polycystic ovary syndrome, patients with acromegaly, patients with NAFLD/NASH, patients with HCV hepatitis, patients with type 2 diabetes, and general population) has produced interesting results, which have led to the hypothesis that the VAI could be considered a marker of adipose tissue dysfunction. Unfortunately, in some cases, on the same patient population, there is conflicting evidence. We think that this could be mainly due to a lack of knowledge of the application limits of the index, on the part of various authors, and to having applied the VAI in non-Caucasian populations. Future prospective studies could certainly better define the possible usefulness of the VAI as a predictor of cardiometabolic risk.

## 1. Introduction

The BMI is wrongly considered a satisfactory predictor of the percentage of body fat, and it is known that it shows a curvilinear and not a linear association with the body fat percentage in both men and women [1].

Beyond the criticisms recently leveled [2] towards the normalization of weight by the square of the height (normalization suggested in the 19th century by Adolphe Quetelet), many factors affect the relationship between BMI and body fat percentage, such as gender, race, high muscle mass (e.g., subjects who practice body building), and changes in hydration status (in particular subjects having retention of extracellular fluids may lead to significant mistakes in interpretation about BMI). In older people, significant changes also occur in both BMI numerator and denominator.

Also, in 2005 in JAMA Katherine Flegal published one of the first studies [3] to analyze in detail the correlation between BMI and all causes of mortality on a large series. This study, based on National Health and Nutrition Examination Survey (NHANES) data, showed that BMI is not a good predictor of mortality risk. A subsequent meta-analysis published in* Lancet *in 2006 [4], evaluating 40 epidemiological studies for a mean followup of 3.8 years, confirmed Flegal's data. These data led in 2006 to an editorial [5] published in Lancet, provocatively titled “Should we continue to use BMI as a cardiovascular risk factor?" which states that “the BMI may now be withdrawn permanently as a clinical or epidemiological tool for evaluation of cardiovascular risk in both primary and secondary prevention.”

This is a condition which deep-down does not appear desecrating to those who are concerned with prevention of cardiovascular disease: in fact the BMI does not appear in any of the versions of the algorithm of Framingham, one of the strongest tools for defining coronary and cardiovascular risk. Indeed, in the Framingham study it was assumed that there could be “metabolically healthy obese subjects.”

An important contribution to evaluation of the influence of obesity on cardiovascular risk is the Interheart Study [6], which shows inconvertible evidence that abdominal obesity makes a higher contribution than BMI to the probability of these events. The following year, focusing attention on the relation between obesity and heart attack risk, Yusuf et al. published a study [7] defining the results more accurately, showing that the association between abdominal adiposity and coronary heart disease risk is highly significant in all geographical areas in which Interheart Study data were collected.

These data incontrovertibly seem to support the usefulness of the evaluation of waist circumference (WC) in cardiovascular risk stratification, having been considered the most valid index of regional distribution of adipose tissue. Measurement of body circumferences, although it is a valid method, requires an accurate performance method in order to provide reproducible information. Several studies have shown that WC is strongly related to visceral fat and abdominal adiposity, more than BMI and waist/hip ratio.

The only limitation of WC is inaccurate distinction between visceral and subcutaneous adipose tissue in the abdominal region.

## 2. A New Method for Evaluation of Adipose Distribution and Function (Visceral Adiposity Index)

**Figure 1:**
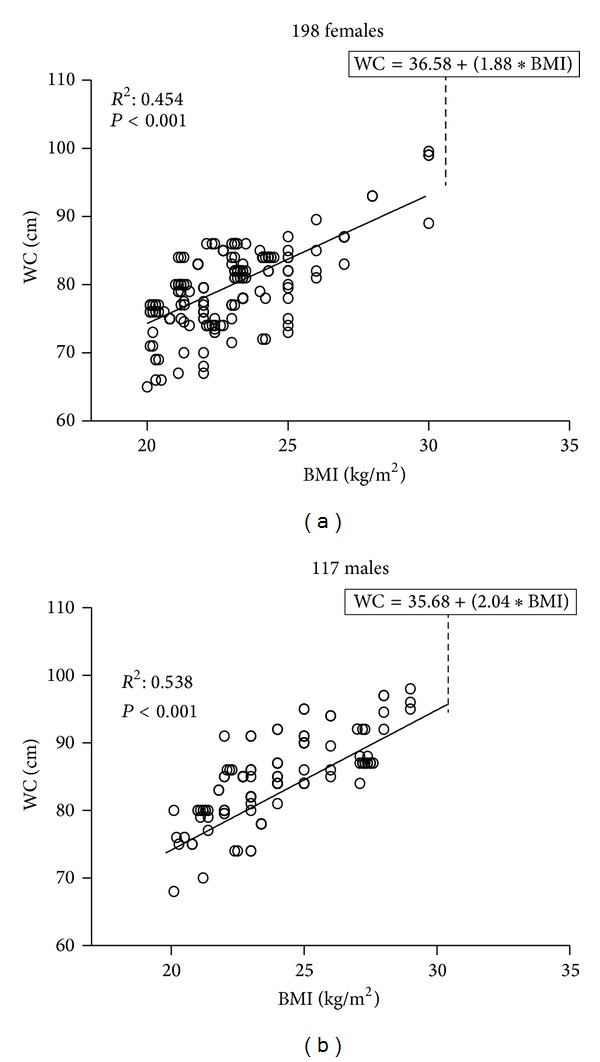
Linear relationship observed between BMI and WC in 315 primary care patients, with BMI between 20 and 30 Kg/m^2^ and age 43.46 ± 14.30 years (range 19–83), selected because of absence of diabetes mellitus or FPG ≥ 5.6 mmol/L, high blood pressure, dyslipidemia, metabolic syndrome and cardiovascular disease (CVD). A model of adipose distribution (MOAD) was created based on this gender-specific linear equation. Taken from Amato et al. [8].

The Visceral Adiposity Index (VAI) is an empirical-mathematical model, gender-specific, based on simple anthropometric (BMI and WC) and functional parameters (triglycerides (TG) and HDL cholesterol (HDL)), and indicative of fat distribution and function [8]. It is an empirical-mathematical model that does not originate from theoretical assumptions, but from observation in a healthy normal/overweight population of a linear relationship between BMI and CV, from which a linear equation has been extrapolated (Figure 1).

At first a model of adipose distribution (MOAD) was created based on this linear equation (which shows a strong correlation with visceral fat mass determined by MRI. Subsequently MOAD was corrected for triglyceride and HDL cholesterol levels, determining the VAI:

(1) Females: VAI=(WC36.58+(1.89×BMI)) ×(TG0.81)×(1.52HDL),Males: VAI=(WC39.68+(1.88×BMI)) ×(TG1.03)×(1.31HDL),

where WC is expressed in cm, BMI in K/m^2^, TG in mmol/L, and HDL in mmol/L.

The VAI has shown a strong positive correlation with peripheral glucose utilization during euglycemic hyperinsulinemic clamp and seems to be independently associated with cardio- and cerebrovascular events [8]. In the last three years, it has been reported on more than 30 publications, in which the capability of the VAI to express a possible “adipose tissue dysfunction” and the cardiometabolic risk associated to it have been evaluated.

## 3. VAI in the General Population as a Marker of Cardiometabolic Risk

The main aim of our research field on the VAI has been to identify a simple clinical marker of adipose tissue dysfunction (indirectly reflecting cardiometabolic risk), before it develops into an overt metabolic syndrome and/or a cardiovascular complication. Unfortunately, today there are still no long-term prospective studies that allow us to evaluate the predictive power of the VAI regarding cardiovascular risk.

Our first study on the VAI [8], in a population of 1,498 Caucasian primary care patients, already showed that there was a strong independent association with both cardiovascular (odd ratio (95% CI): 2.45 (1.52–3.95)) and cerebrovascular events (odd ratio (95% CI): 1.63 (1.06–2.50)); in the same study, a receiver operating characteristic (ROC) analysis proved greater sensitivity and specificity of VAI, compared to its individual components (WC, BMI, HDL, and TG) with regard to cardiovascular and cerebrovascular events. In another ROC analysis on 1518 Peruvian adults, in which various measures of adiposity were evaluated, VAI, WC, and waist-height ratio (WHtR) were the best predictors of the individual components of the metabolic syndrome [9]. In particular, the VAI showed good predictive power regarding the visceral adiposity-related risk of type 2 diabetes [10–12] and hypertension [13]. Although in some studies the increase in VAI was significantly associated with a significant reduction in insulin sensitivity [8, 14, 15], VAI is not to be seen as an index of insulin sensitivity, but as an indicator of altered adipose function which is associated with insulin resistance.

Although the VAI was modelled on a Caucasian population, several studies confirm the validity of its use with other races. For example, in a large case-control study, a high VAI is associated with elevated risk of CHD in Chinese men and women [16]. Moreover, in a large cross-sectional study on 1,764 primary care patients, through an ROC analysis, appropriate stratified-for-age cut-offs were identified that were able to identify a supposed adipose tissue dysfunction [17] (Table 1). These cut-offs have been more or less confirmed in a recent study (data not yet published) in which adipose tissue dysfunction was directly investigated through a large panel of proinflammatory adipokines.

Despite this suggestive evidence, there are also conflicting data like those shown by an Iranian study, which concluded that using the “complex” VAI, instead of other simple anthropometric measures, may lead to loss of much information needed for predicting incident CVD [18]. Another Canadian study also indicated the nonsuperiority of the VAI, compared to BMI and WC, in predicting the visceral adipose tissue change in postmenopausal women during a low-calorie diet [19]. These data suggest the need for further prospective studies which take into account the greater variability in the years of the VAI, compared to other simple anthropometric measures.

## 4. VAI in NAFLD and NASH

In the hepatological field, the VAI has been investigated in several studies in patients with NAFLD, with the main objective of identifying a clinical marker predictive of evolution towards necroinflammatory injury and fibrosis [20–25]. In this respect, there are contrasting results between the various studies, since according to some authors the VAI accurately predicted progressive liver histology more accurately than other validated noninvasive scores and identified patients with NAFLD at increased CVD risk [20, 24, 25], while according to other authors [21–23] the VAI is not more powerful than other anthropometric indices in discriminating steatosis from steatohepatitis. In our opinion, these discrepancies are attributable to differences between the patients enrolled, especially concerning the variables included in the VAI. This especially applies (as explained in the proper use and limits section) to the mean of triglyceride levels in the various populations [26]. Moreover, in the hepatological field, an interesting result was obtained from a study [27] on patients with chronic hepatitis C due to genotype 1. In these patients only older age, high VAI, and fibrosis were independently associated with moderate-severe necroinflammatory activity by a logistic regression analysis; a higher VAI also has a direct correlation with viral load. Probably, adipose tissue dysfunction (indirectly expressed by the VAI) by way of free fatty acid and proinflammatory cytokine secretion could directly participate in both liver steatosis and induction of inflammation. In this complex interplay between the liver and adipose tissue, HCV could have an important role. It is possible not only that adipose tissue could provide fatty substrates and a proinflammatory status, favouring HCV replication, but also that HCV could interfere with adipocyte function indirectly by increasing the inflammatory status and directly by colonizing adipocytes or immune cells infiltrating adipose tissue [28, 29].

## 5. VAI in Various Endocrine Diseases

It is now known that carbohydrate, lipid, and protein metabolism and the whole cardiovascular system respond to multiple endocrine signals, including those originating from the adipose endocrine organ. Therefore, there are several endocrine diseases that expose the patient to a significant cardiometabolic risk. This risk is unfortunately not easily identifiable through the classic systems of valuation (risk charts, Framingham risk score, etc.) based on classical cardiovascular risk factors. Nor, in this field, can one rely on the presence of obesity; it is now known that endocrinopathy may increase cardiovascular risk even in lean subjects. Therefore, especially in young patients with endocrine disease, who have not yet developed an overt metabolic syndrome, the application of the VAI could give useful information.

### 5.1. VAI in the Polycystic Ovary Syndrome (PCOS)

Insulin resistance and abdominal obesity are common but not universal features of PCOS, and they are not always associated with an increased BMI. Indeed, many studies have shown that both lean and obese women with PCOS have insulin resistance [30], though recently it has been shown that in PCOS there is an intrinsic IR that is further worsened by increasing BMI [31]. It is also known that obesity is not necessarily an expression of cardiometabolic risk, given that there exists “metabolically healthy obesity” in which the particular gynoid distribution of fat does not confer a cardiometabolic risk [32]. According to these findings, in women with PCOS, it is necessary to identify a simple clinical index able to distinguish metabolically healthy polycystic ovary syndrome (MH-PCOS) from metabolically unhealthy PCOS (MU-PCOS). In first study of ours [33] it was found that the oligomenorrhoic phenotypes of PCOS (applying the Rotterdam criteria) were characterized by a high VAI and a condition of cardiometabolic risk. Recently in young Korean women with PCOS, the VAI positively correlated with the visceral fat area (measured with computed tomography) and visceral-to-subcutaneous fat ratio and negatively correlated with the insulin-mediated glucose utilization (M value) during euglycemic hyperinsulinemic clamp [14]. Another recent study shows that in women with PCOS the VAI increases in relation to the severity of anovulation, insulin resistance, and inflammation [34]. Recently these findings have led us to verify whether it was possible to distinguish women with MH-PCOS from women with MU-PCOS through the use of simple diagnostic tools such as BMI, waist to hip ratio (WHR), the at-risk category suggested by Androgen Excess Society (AES) [35], and the VAI. Despite the risk according to AES, a BMI > 27 kg/m^2^ and a VAI > 1.675 have similar diagnostic value in detecting adverse metabolic profile in women with PCOS, a risk that according to AES and BMI > 27 kg/m^2^ criteria tends to overestimate the problem; therefore, given the simplicity of WC and BMI measurement and TG and HDL assessment, it has been suggested that the VAI could be an easy and useful tool for the assessment of MU-PCOS in daily clinical practice and in population studies [36].

### 5.2. VAI in Acromegaly

Untreated acromegalic patients have a decreased fat mass and increased lean body mass due to the lipolytic effect of GH [37, 38]. In such patients the subcutaneous adipose tissue is reduced, but the visceral adipose tissue secretes a number of cytokines that may cause adipocyte dysfunction and contribute to insulin resistance in the liver and skeletal muscle and adversely affect pancreatic *β*-cell function [39]. This pathophysiological condition can escape the dichotomous criteria of metabolic syndrome, especially because of the absence in many cases of an increased waist circumference. Although the VAI is a gender-specific index (separately modelled on healthy women and healthy men), in women with active acromegaly it is strongly associated with insulin resistance, adipose tissue dysfunction, and cardiometabolic risk, especially in the postmenopausal age [40]. In another study [41] the VAI also appears to be associated with disease activity, inversely with adiponectin levels, insulin sensitivity, and insulin secretion, and independently correlated with GH levels. These data suggest that the VAI could therefore be used as a useful tool for the assessment of cardiometabolic risk associated with active acromegaly, especially in postmenopausal acromegalic women.

### 5.3. VAI in Prolactinoma

Much is known about the effects of prolactinomas on the reproductive system, but few data are yet available regarding metabolism and adipose tissue function. However, in both men and women alterations have been observed in the distribution of adipose tissue, probably related to chronic hyperprolactinemia [42, 43]. In two recent studies it has been shown that in patients with prolactinoma cabergoline treatment is able to significantly reduce the VAI and improve metabolic profile and insulin sensitivity [44, 45]. In clinical practice, in young patients with prolactinoma without overt metabolic involvement, the VAI could be useful both at diagnosis and during the treatment followup, to prevent the metabolic complications of the disease.

### 5.4. VAI in Cushing Disease

The application of VAI in overt Cushing's syndrome, on the basis of the typical clinical and phenotypic alterations of the disease, is probably not very useful. The fact is that almost all patients with overt Cushing have a pathological increase in waist circumference, BMI, and triglycerides and lower HDL cholesterol. In these patients (who for the reasons indicated above present a high VAI), the use of the index adds nothing to simple application of the metabolic syndrome criteria. Furthermore, if on the one hand the VAI is probably an indicator of adipose dysfunction, on the other hand it is known that all patients with Cushing have visceral fat dysfunction. In our opinion, the only application field of the VAI in Cushing's syndrome is epidemiological studies with a high sample size, taking into account the well-known limits of the index. In a recent study, the VAI was tested in 140 patients with Cushing's syndrome [46]; in this study women with Cushing showed a significantly higher VAI (considering that in men with Cushing the VAI was also high, compared to values observed in the general population), consequent of the influence of cortisol excess on visceral adipose dysfunction. The increase in the VAI, specifically described in women with Cushing, confirms the loss of gender-related cardiovascular protection of the women when they develop an increase in visceral adipose tissue, as also observed in other endocrine diseases, such as acromegaly and prolactinoma [40, 44]. On the basis of these experiences, even if we consider unnecessary the use of the VAI in case-control studies on Cushing patients versus healthy populations, this index could show its usefulness in prospective studies which contemplate therapeutic outcomes.

## 6. Proper Use and Limits of Visceral Adiposity Index

The scientific evidence of the last three years on the use of VAI has allowed us to understand the usefulness of the index and especially also its limitations. These have been summarized in a recent letter to the journal NMCD [26]. The main aspect to consider is that the VAI is an indicator of early cardiometabolic risk in all borderline conditions in which overt metabolic syndrome is not present. This is explained by the fact that three of the variables making up the VAI (WC, TG, and HDL) are dichotomically expressed in the criteria for metabolic syndrome. Also, of the four variables that make up the VAI, triglycerides present the problem of the wide range of values found in the general population, and waist circumference the problem of the validity of measurement in subjects with morbid obesity and pendulous abdomen. For these reasons it has been recommended in an individual patient or in small sample studies the VAI should not be applied, above all in the presence of morbid obesity, pendulous abdomen, severe hypertriglyceridemia, and/or use of fibrates [26]. In this regard, we observed that, above values of serum triglycerides > 3.15 mmol/L (279 mg/dL), the impact of triglyceridemia on the VAI becomes very crucial: in these cases triglycerides alone can provide us with much more information than the VAI regarding the cardiometabolic risk associated with adipose dysfunction. The same can also be said about subjects with morbid obesity, in which even WC has no great diagnostic value. An example of incorrect application of the VAI is a recent study in a population with obstructive sleep apnoea, where the authors wanted to test the association with the severity of sleep apnoea [47]. In actual fact, in many patients with this problem morbid obesity and a metabolic syndrome occurred.

Another important aspect that deserves to be investigated concerns changes in the VAI with a low-calorie diet. Theoretically a healthy weight loss achieved with a mildly reduced calorie-balanced diet (which does not result in intense and rapid lipolysis), possibly accompanied by aerobic physical activity, should result in a reduction in the VAI. This aspect has not yet been studied. An original recent study found that Ramadan fasting in healthy adult men was associated with significant decreases in body weight, BMI, waist circumference to height ratio (WHtR), and Body Adiposity Index (BAI), but we found no significant changes in the VAI [48]. These data indicate that this particular form of fasting for a period of about a month, even if it determines weight loss, does not reduce the cardiometabolic risk.

Another important limitation to consider is the application of the VAI in non-Caucasian populations and in patients aged less than 16 years. The fact is that the numerical factors of the index arise from a mathematical modelling process on healthy Caucasian men and women, aged between 19 and 83 years. In this regard a study was recently published which evaluated the VAI in children [49]. The authors rightly conclude that the VAI should be extrapolated with caution in this age range. We will add more: the VAI “absolutely should not be applied in this age range,” because the numerical factors that make up the formula of the VAI are derived by the linear equation linking the BMI and waist circumference in a healthy adult population and mean levels of triglycerides and HDL cholesterol in the same population (Online-Only Appendix in [8]).

Having made these considerations, we recommend the application of VAI in the following populations: healthy or apparently healthy population with BMI < 40 kg/m^2^, patients with one or two of the 5 components of the metabolic syndrome, women with PCOS, and patients with endocrine disorders (e.g., acromegaly, adult GH deficiency, hypogonadism, hyperprolactinemia, or abnormal thyroid function).

## 7. Conclusion

An appropriate use of the VAI (which necessarily implies absolute knowledge of what we have already defined as limits of application) could help us in various clinical situations to assess the complex phenomenon of “adipose tissue dysfunction," especially in the absence of an overt metabolic syndrome. Unfortunately, today the ability of the VAI to express adipose tissue function is only to be seen as a hypothesis; indeed few studies exist that have evaluated the correlation between it and adipocytokine production [11, 25, 41]. However, in our recent study [50] we evaluated the correlations among various anthropometric indices (BMI, waist circumference (WC), hip circumference (HC), waist/hip ratio (WHR), BAI, and Visceral Adiposity Index (VAI)) and several adipocytokines (visfatin, resistin, leptin, soluble leptin receptors (sOB-R), adiponectin, ghrelin, adipsin, PAI-1, vascular endothelial growth factor (VEGF), hepatocyte growth factor (HGF) TNF-*α*, hs-CRP, IL-6, and IL-18) in patients with type 2 diabetes. Our data suggest that the VAI, among the most common indexes of adiposity assessment, would be an easy tool for clearly mirroring adipose tissue dysfunction and its associated cardiometabolic risk.

In conclusion, although still lacking prospective studies that can attribute a prognostic role to the VAI regarding cardiovascular risk, given the simplicity of WC and BMI measurement and triglycerides and HDL cholesterol assessment, we suggest that the VAI would be an easy tool for the evaluation of adipose tissue dysfunction and its associated cardiometabolic risk in various patient populations, mainly in the absence of an overt metabolic syndrome.

## Figures and Tables

**Table 1:** Age-stratified cut-off points of VAI for identification of adipose tissue dysfunction (ATD).

	ATD absent	Mild ATD	Moderate ADT	Severe ADT
Age < 30 years	≤2.52	2.53–2.58	2.59–2.73	>2.73
≥30 < 42 years	≤2.23	2.24–2.53	2.54–3.12	>3.12
≥42 < 52 years	≤1.92	1.93–2.16	2.17–2.77	>2.77
≥52 < 66 years	≤1.93	1.94–2.32	2.32–3.25	>3.25
≥66 years	≤2	2.01–2.41	2.42–3.17	>3.17
